# Reduction in the diagnostic interval after the introduction of cancer patient pathways for colorectal cancer in northern Sweden

**DOI:** 10.1080/02813432.2023.2234003

**Published:** 2023-07-14

**Authors:** P. Fjällström, C. Hörnsten, M. Lilja, C. Hultstrand, A. B. Coe, S. Hajdarevic

**Affiliations:** a Department of Nursing, Umeå University, Umeå, Sweden; b Department of Clinical Sciences, Psychiatry, Umeå university, Umeå, Sweden; c Department of Public Health and Clinical Medicine, Unit of Research, Education, and Development, Östersund Hospital, Umeå University, Umeå, Sweden; d Department of Sociology, Umeå University, Umeå, Sweden; e Department of Public Health and Clinical Medicine, Family Medicine, Umeå University, Umeå, Sweden

**Keywords:** Colorectal cancer, cancer patient pathways, diagnostic interval, primary healthcare, socioeconomic factors, symptoms, time to diagnosis

## Abstract

**Objective:**

To compare the diagnostic interval for patients with colorectal cancer before and after the introduction of cancer patient pathways in northern Sweden.

**Design:**

A retrospective study comparing two cohorts (2012 and 2018) of patients diagnosed with colorectal cancer before and after the introduction of cancer patient pathways in 2016.

**Setting:**

Three counties in northern Sweden with large sparsely populated areas and some cities (637143 residents ∼5.1 residents/km^2^).

**Subjects:**

Patients were included from the Swedish Cancer Register. Electronic health records reviews were performed and linked to socioeconomic data from Statistics Sweden.

**Main outcome measures:**

Differences in the diagnostic intervals, the patient intervals and the characteristics associated with the longest diagnostic intervals and investigations starting at the emergency department.

**Results:**

The two cohorts included 411 patients in 2012 and 445 patients in 2018. The median diagnostic interval was reduced from 47 days (IQI 18–99) to 29 days (IQI 9–74) (*p* < 0.001) after the introduction of cancer patient pathways in general. Though for the cases of cancer in the right-side (ascended) colon, the reduction of the diagnostic interval was not observed and it remained associated with investigations starting at the emergency department.

**Conclusion:**

Our results indicate that cancer patient pathways contributed to an improvement in the diagnostic interval for patients with colorectal cancer in general, yet not for patients with cancer in the right-side colon.

**Implication:**

In general, cancer patient pathways seem to reduce the diagnostic interval for colorectal cancer but it is not a sufficient solution for all colorectal cancer localisations.

## Key points

Diagnostic interval for colorectal cancer reduced in general, particularly for patients seeking primary healthcare, after the introduction of cancer patient pathways.

Patients with cancer in right-side colon still have long diagnostic intervals and mainly start their investigation through the emergency department.

## Introduction

Colorectal cancer (CRC) is the third most common cancer in Sweden and globally [1,2]. Patients with CRC commonly start to seek care in primary healthcare, presenting both bowel-specific and non-specific symptoms, thus making CRC hard to discern [3,4]. Adding to the complexity, CRC located in the right-side colon often presents with non-specific symptoms that are difficult to identify as an indication of cancer and the clinical suspicion is less often raised, why it is often diagnosed in an advanced stage [5,6]. Patients typically experience such non-specific symptoms for a long time before contacting a healthcare provider [7].

Researchers have sought to identify socioeconomic factors affecting the prognosis, survival and time to diagnosis of CRC. Short education [8] and low income [9,10] as well as long-distances to healthcare [11] are found to be associated with poorer survival and advanced tumour stages in CRC. Furthermore, others have found that longer diagnostic time intervals are related to being female, while there are inconsistent results regarding factors such as age, income and education [12]. However, timely diagnosis is crucial since advanced stages of CRC are related to a worse prognosis [13] and better knowledge of reforms and tools adopted within healthcare systems to shorten time to diagnosis is needed [14].

In several European countries as well as in Sweden, Cancer Patient Pathways (CPP) have been introduced as a tool to shorten the time to diagnosis and reduce inequality in cancer care [15–19]. The specific CPP for CRC in Sweden was introduced in 2016. The diagnostic interval (DI) is defined as the time from the first symptom presented in healthcare to diagnosis [20]. Studies examining DI for CRC after the introduction of CPPs are scarce. Some studies indicate that CPPs reduce DI [16] and might improve cancer prognosis [15]. However, CPPs may also prolong the time to diagnosis for patients with vague non-specific symptoms [21]. Knowledge is limited on the potential influence of CPPs on time to diagnosis for people living in a sparsely populated area, such as northern Sweden, as well as in relation to sociodemographic characteristics of the population.

## Aim

This study aimed to compare the diagnostic interval for patients with colorectal cancer before and after the introduction of cancer patient pathways in sparsely populated counties in northern Sweden.

## Methods

This was a retrospective observational study comparing two cohorts from three counties in northern Sweden, specifically Jämtland Härjedalen, Västerbotten, and Västernorrland. The first cohort (2012 cohort) included patients diagnosed with CRC between January 2012 and December 2012, while the second cohort (2018 cohort) included patients diagnosed with CRC between July 2017 and June 2018. The STROBE checklist for cross-sectional, case-control and cohort studies has been used as a guide [22].

### Setting

The Swedish healthcare system is publicly funded and has a decentralised structure with twenty-one counties providing healthcare services autonomously through hospitals and primary healthcare units, with some private healthcare providers [23]. Inhabitants decide themselves from which primary healthcare unit they want to receive healthcare. Primary healthcare is most often patients’ first contact and the main gateway for accessing cancer care. None of the three counties had CRC screening programs before the year of 2022. To our best knowledge, no other reforms to improve DI have been implemented between 2012 and 2018 in these counties.

Northern Sweden has both large sparsely populated areas (≥45 min by car to urban areas) and some cities [24]. The included counties in our study had a mean of 637 143 residents within an area of 125 158 km^2^ (5.1 residents/km^2^). The number of units and hospitals differed between counties in 2021: The County of Jämtland Härjedalen had 26 primary healthcare units and one hospital; Västerbotten had 38 primary healthcare units and three hospitals; and Västernorrland had 31 primary healthcare units and three hospitals.

### Study population and data collection

Data were collected from the Swedish cancer register, with a national coverage rate of >98% reported cases [25], on patients diagnosed with CRC for the two cohorts. Inclusion criteria in these cohorts were patients diagnosed with primary CRC (tumour *in situ* excluded) within the included counties. Four cases with tumour in the appendix were excluded because these are clinically different from other CRCs and often identified during complicated circumstances. Hence, roughly 97% of patients diagnosed with CRC in the investigated geographical areas and time span from the Swedish cancer register were included. A power analysis based on an ability to detect a ten days’ difference between cohorts was conducted before data collection showing a minimum need of 320 patients in each cohort (power 80%, α 5%). The final cohorts included 411 patients from 2012 and 445 patients from 2018. Google maps were used to generate the distance from the patients’ homes to the closest hospital. Next, a physician with experience within the field in each county reviewed the electronic health record (EHR), which includes patients’ personal identifiers, and all contacts with both primary and secondary healthcare. Data on patient appointments and dates during a span of a minimum of two years, and further back if needed, concerning the processes of diagnosis of CRC were retrieved. The EHR reviews were structured according to a predefined form and first contact with a physician was defined retrospectively from the EHRs as the first presentation of any symptoms related to the individual’s CRC. Observed symptoms within the interval from patients’ first contact to the referral to secondary care included blood in stool, bowel pain, weight loss, fatigue, changed bowel- and stool habits, and only in 2018 anaemia. Additionally, a few incidental radiological findings led to a CRC diagnosis with a DI of zero days. The documented date of perceived first symptom before seeking care was treated as a start of patient delay. Documented dates of healthcare appointments during the CRC investigation in each county were collected by reviewing the EHR shared by primary and secondary healthcare. Four primary healthcare units, with a total of approximately 16000 listed inhabitants within one urban setting, used a separate EHR system and were not part of the inclusion process. Finally, this data was linked to data on socioeconomic factors (education and family income) from Statistics Sweden, the longitudinal integration database for health insurance and labour market studies.

### Variables

In our study, the outcome variable DI [20] was defined as the time in days from the first appointment with a physician to CRC diagnosis. However, in Sweden it is usually a nurse who has the first contact with patients in primary healthcare, thus, we also examined the dates from first contact, regardless of healthcare professional, to diagnosis. The patient interval is defined as the time from the first symptom to the first presentation of symptom in healthcare [20]. Finally, the outcome variable for patients with the longest DI was dichotomised and defined as the 20% with the longest DI in each year, i.e. DI ≥125 days in 2012 (reference DI <125 days) and DI ≥91 days in 2018 (reference DI <91 days).

Sociodemographic variables were dichotomised, except for age, and defined as following: *sex* (male and female); *age*, divided into age groups (according to quartiles) 64 years and younger, 65–72 years, 73–79 years and 80 years and older; *distance to the hospital* as long-distance (the 20% with the longest distance to the hospital, ≥57 kilometres) and shorter distance (<57 kilometres); *education* as shorter education (completed elementary school or high school education) and longer education (completed college or university education); *available family income* into lower economic standard (below 60% of median in Sweden each year [26], <137300 SEK ≈ <12187 €in 2012 and <153200 SEK ≈ <14714 €in 2018) and higher economic standard (all others in each cohort).

Additional variables of the study were the localisation of the tumour, CRC symptoms, CRC stage, first contact with healthcare, and county. The *localisation of the tumour* variable was dichotomised into CRC right-side (ascending colon) and CRC all others (transverse colon, descending colon, sigmoid colon, rectum, and unspecified). The *CRC symptoms* were dichotomised, according to the Swedish CPP program from 2016, with CPP-qualifying symptoms (blood in stool and/or changed bowel habits) and without CPP-qualifying symptoms (e.g. weight loss, fatigue, and bowel pain). Unfortunately, data collected in 2012 did not include anaemia and thus is not included as a CPP-qualifying symptom in the comparison between cohorts. For the comparison, 20 cases (4.6%) from 2018 had anaemia as a single CPP symptom. The *tumour stage* variable was based on tumour, node, and metastasis (TNM) categorisation [27]. Two counties had incomplete data concerning stages in the cohort from 2012. Due to this, we could only compare the cohorts in the county of Jämtland Härjedalen which had complete data regarding stages. The *first contact with healthcare* variable was dichotomised into primary healthcare (primary healthcare units) and secondary healthcare (emergency department and hospital wards).

### Analysis

In this study, we mainly focused on descriptive statistics to illustrate differences in DI by comparing the two cohorts diagnosed with CRC before (2012) and after the introduction of CPPs (2018). The outcome variables (DI and patient interval) had a positively skewed distribution, and all other data were skewed except for the age variable. We therefore mostly used non-parametric methods in our analysis, after assessing the normality of the data with a visual examination of histograms and using Skewness-Kurtosis test. The Pearson’s Chi-square test was used on categorical variables and the Mann-Whitney U test on continuous variables to compare the cohorts in general as well as within sociodemographic groups and other patient characteristics. Therefore, the median of DI was reported as well as an interquartile interval (IQI) and range for each of the independent variables. Next, the patient interval was analysed in general, and in relation to sex and age, and reported with median and IQI. Finally, we used logistic regression, including potentially relevant variables, to analyse the association of patient characteristics with the longest DI as well as those with acute initiated investigations in each cohort and presented with an odds ratio (OR) and confidence interval (CI).

All analyses were performed using IBM SPSS Statistics (version 27) and alpha was defined at the statistical significance level of p-value ≤0.05 with a 95% confidence interval. All analyses were performed at a secure platform provided by Statistics Sweden.

## Results

This study included 411 patients from 2012 and 445 patients from 2018 diagnosed with CRC. The patient characteristics are presented in Table 1. Data on DI was observed for 410 patients (99.8%) 2012 and 434 patients (97.5%) 2018 respectively. In the analysis of the patient interval, data was observed for 221 patients (53.8%) in 2012 and 301 patients (67.3%) in 2018.

**Table 1.** Characteristics of patients diagnosed with colorectal cancer (CRC) in 2012 and 2018.

	Cohort 2012 (*n* = 411)				Cohort 2018 (*n* = 445)				Comparison 2018–2012
	*n* (%)	Mean/ Median	CI/ IQI	Range	*n* (%)	Mean/ Median	CI/ IQI	Range	*p*-value
Age (mean value)	411 (100)	71.3	70.2–72.5	28–95	445 (100)	72.3	71.3–73.3	37–93	0.275
≤64 years	103 (25.1)				86 (19.3)				0.092
65–72 years	90 (21.9)				125 (28.1)				
73–79 years	110 (26.8)				118 (26.5)				
≥80 years	108 (26.3)				116 (26.1)				
Sex	411 (100)				445 (100)				
Female	191 (46.5)				193 (43.4)				0.399
Male	220 (53.5)				252 (56.6)				
Education	409 (100)				428 (100)				
Shorter education	333 (81.4)				325 (75.9)				0.064
Longer education	76 (18.6)				103 (24.1)				
Available family income (SEK/year, median value)	411 (100)	236 000	139 100 − 359 500	−223 100 − 3 735 000	431 (100)	279 200	172 800 − 417 000	−600 − 8 625 800	
Lower economic standard	94 (22.9)				82 (19)				0.198
Higher economic standard	317 (77.1)				349 (81)				
Distance to the hospital (kilometers, median value)	411 (100)	15	4–49	0– 264	445 (100)	18	5–49	1–248	0.301
Longer distance	81 (19.7)				89 (20)				0.983
Shorter distance	330 (80.3)				356 (80)				
County	411 (100)				445 (100)				
Västernorrland	143 (34.8)				158 (35.3)				0.489
Jämtland Härjedalen	84 (20.4)				104 (23.4)				
Västerbotten	184 (44.8)				184 (41.3)				
First contact with healthcare	411 (100)				445 (100)				
Primary healthcare	314 (76.4)				320 (71.9)				0.156
Secondary healthcare	97 (23.6)				125 (28.1)				
CRC symptoms	408 (100)				434 (100)				
With CPP-qualifying symptoms *	280 (68.6)				257 (61.9)				
Blood in stool	153 (37.5)				164 (38.8)				
Changed bowel habits	227 (55.6)				185 (43.5)				
Anaemia	^a^				182 (42.4)				
Without CPP-qualifying symptoms	128 (31.4)				158 (38.1)				
Weight loss	84 (20.7)				88 (20.8)				
Fatigue	107 (26.3)				126 (29.6)				
Bowel pain	146 (35.9)				170 (40)				
TNM staging ^b^	^b^				426 (100)				
Unknown	^b^				11 (2.6)				
I	^b^				76 (17.8)				
II	^b^				126 (29.6)				
III	^b^				129 (30.3)				
IV	^b^				84 (19.7)				
Tumour localization	411 (100)				445 (100)				
CRC right side	148 (36)				142 (31.9)				0.22
CRC all others	263 (64)				303 (68.1)				

Values presented are means with confidence intervals (CI) or median values with interquartile intervals (IQI). Incomplete data are presented with ^a^ or ^b^.

**Age** – Divided into quartiles and presented in age groups.

**Shorter education** - Completed elementary school or high school education. **Longer education** - Completed college or university education.

**Lower economic standard** - Available family income in Swedish krona (SEK) / year, below 60% of median nationally each year, specifically <137300 SEK (≈ <12 187 €) in 2012 and <153200 SEK (≈ <14 714 €) in 2018. **Higher economic standard** - Above Low economic standard in each cohort.

**Longer distance** - The 20% with the longest distance to the hospital (57–264km). **Shorter distance** - <57 km to the nearest hospital.

**Primary healthcare** - initial contact in primary healthcare units. **Secondary healthcare** - initial contact in the emergency department or hospital wards.

**With CPP-qualifying symptoms** – Anaemia, blood in stool and changed bowel habits. ^a^Data regarding anaemia is missing in cohort 2012 and thus not included as a CPP-qualifying symptom in any of the cohorts. **Without CPP-qualifying symptoms** – E.g., bowel pain, fatigue, and weight loss. The proportions in each symptom are divided into whether the symptom was presented or not.

**Tumour, node, and metastasis (TNM) staging**. ^b^Two counties had incomplete data of documented remote metastasis in the 2012 cohort. We only compared the cohorts in the county (Jämtland Härjedalen) with complete data and the cohorts were comparable though too small to analyse further.

**CRC right side** - Ascending colon. **CRC all others** - Transverse colon, descending colon, sigmoid colon, rectum, and unspecified location.

### Reduction in time to diagnosis

**Figure 1.**
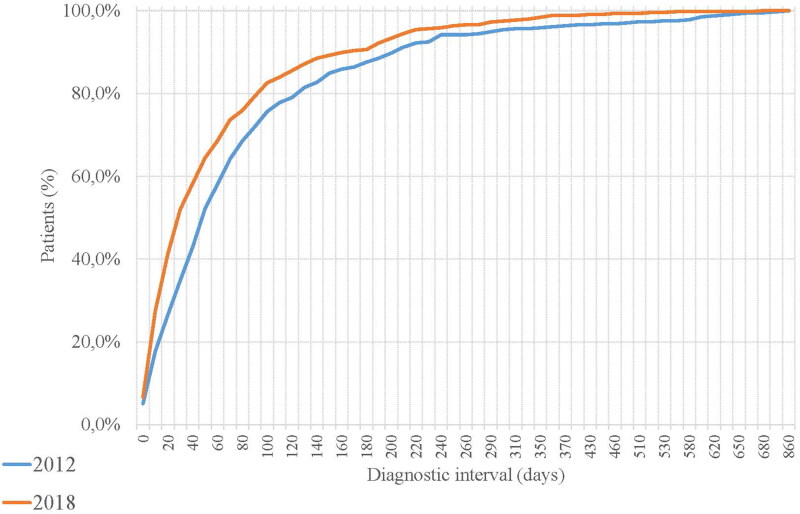
Cumulative curve, proportion of patients in relation to diagnostic interval (DI) in each cohort.

Figure 1 and Table 2 demonstrate a comparison of DI in CRC between the two cohorts overall and stratified. In total, the median DI was reduced by 18 days in 2018 compared to 2012 and this reduction was seen irrespective sex and for most age groups (see, Table 2). Moreover, the 75 percentile of DI decreased in 2018, which means that less patients had a very long DI. In addition, the DI in 2018 was reduced by approximately two weeks for patients diagnosed in primary healthcare, while there was no appreciable difference for patients diagnosed in secondary healthcare. However, when patients’ initial contact in primary healthcare was with a healthcare professional other than a physician, the DI was approximately 5 days longer in both cohorts (further data not presented). The DI was further examined in relation to distance to hospital, education, family income and observed symptoms which indicated a reduction in DI (see Table 2). In the case of patients diagnosed with CRC in the right-side colon, no noticeable reduction was observed. Counties with a longer DI in 2012 show a great improvement in DI in 2018. Additionally, the patient interval (median) between the cohorts reduced from 60 days (IQI 21–180) in 2012 to 30 days (IQI 3–120) in 2018 (*p* < 0.001). Also, among patients 80 years and older, the patient interval was reduced by 15 days (*p* 0.02) in 2018, while for those under 80 years it was reduced by 30 days (*p* 0.003). A similar reduction was observed both for females (30 days, *p* 0.005) and males (15 days, *p* 0.008) (further data not presented).

**Table 2.** Diagnostic interval (DI) in days for patients with colorectal cancer (CRC) compared between the cohorts from 2012 and 2018.

	Cohort 2012 (*n* = 411)				Cohort 2018 (*n* = 445)				Comparison 2018-2012	
	n (%)	Median	IQI	Range	n (%)	Median	IQI	Range	Median difference	*p*-value
**DI total**	410 (100)	47	18–99	0–859	434 (100)	29	9–74	0–673	**−18**	**<0.001**
**Age**	410 (100)				434 (100)					
≤64 years	102 (24.9)	40	16–85	0–577	80 (18.4)	27	7–72	0–673	−13	0.089
65–72 years	90 (22.0)	44	17–98	0–859	123 (28.4)	29	8-63	0–428	**−15**	**0.012**
73–79 years	110 (26.8)	54	19–90	0–695	116 (26.7)	28	10–73	0–456	**−26**	**0.036**
≥80 years	108 (26.3)	62	27–127	0–645	115 (26.5)	39	14–100	0–539	**−23**	**0.025**
**Sex**	410 (100)				434 (100)					
Female	191 (46.6)	55	26–116	0–859	188 (43.3)	39	9–85	0–520	**−16**	**0.002**
Male	219 (53.4)	42	16–84	0–695	246 (56.7)	26	9–64	0–673	**−16**	**0.006**
Difference		13				13				
**Education**	408 (100)				417 (100)					
Shorter education	332 (81.4)	49	20–102	0–695	315 (75.5)	30	11–74	0–539	**−19**	**<0.001**
Longer education	76 (18.6)	41	13–86	0–859	102 (24.5)	24	7–84	0–673	**−17**	**0.101**
Difference		8				6				
**Economic standard**	410 (100)				420 (100)					
Lower standard	94 (22.9)	58	17–125	0–695	81 (19.3)	45	14–98	0–539	−13	0.48
Higher standard	316 (77.1)	46	19–92	0–859	339 (80.7)	27	9–70	0–673	**−19**	**<0.001**
Difference		12				18				
**Distance to hospital**	410 (100)				434 (100)					
Longer distance	81 (19.8)	50	19–99	0–660	89 (20.5)	39	15–77	0–539	−11	0.366
Shorter distance	329 (80.2)	46	18–100	0–859	345 (79.5)	27	8–74	0–673	**−19**	**<0.001**
Difference		4				12				
**County**	410 (100)				434 (100)					
Västernorrland	142 (34.6)	52	22–100	0–660	147 (33.9)	35	11–81	0–673	**−17**	**0.013**
Jämtland Härjedalen	84 (20.5)	37	12–161	0–583	103 (23.7)	28	8–126	0–539	−9	0.28
Västerbotten	184 (44.9)	49	18–124	0–859	184 (42.4)	29	9–84	0–357	**−20**	**0.001**
**First contact with healthcare**	410 (100)				434 (100)					
Primary healthcare	314 (76.6)	52	27–106	0–660	311 (71.7)	33	14-82	0–539	**−19**	**<0.001**
Secondary healthcare	96 (23.4)	22	5–91	0–859	123 (28.3)	19	2–63	0–673	−3	0.107
Difference		30				14				
**CRC Symptoms**	407 (100)				410 (100)					
With CPP-qualifying symptoms *	279 (68.6)	46	18–107	0–859	255 (62.2)	26	9–64	0–539	**−20**	**<0.001**
Without CPP-qualifying symptoms	128 (31.4)	53	21–98	0–695	155 (37.8)	35	8–88	0–428	**−18**	**0.048**
Difference		7				9				
**Tumour localization**	410 (100)				434 (100)					
CRC right side	148 (36.1)	51	22-108	0–695	138 (31.8)	48	14-106	0–520	−3	0.426
CRC all others	262 (63.9)	46	17–98	0–859	296 (68.2)	26	8–65	0–673	**−18**	**<0.001**
Difference		5				22				

Values presented are median values with interquartile interval (IQI) and range.

**Age** – Divided into quartiles and presented in age groups.

**Shorter education** – Completed elementary school or high school education. Longer education – Completed college or university education.

**Lower economic standard** – Available family income in Swedish krona (SEK) / year (below 60% of median nationally each year), specifically <137300 SEK (<12 187 €) in 2012 and <153200 SEK (≈ <14 714 €) in 2018. Higher economic standard – Above Low economic standard in each cohort.

**Longer distance** – The 20% with the longest distance to the hospital (57-264km). Shorter distance – <57 km to the nearest hospital.

**Primary healthcare** – Initial contact in primary healthcare units. Secondary healthcare – Initial contact in emergency department or hospital wards.

**With CPP-qualifying symptoms** – Blood in stool and changed bowel habits. *Data regarding anaemia is missing in cohort 2012 and thus not included as a CPP-qualifying symptom in any of the cohorts. Without CPP-qualifying symptoms – e.g. bowel pain, fatigue and weight loss.

**CRC right side** – Ascending colon. CRC all others – Transverse colon, descending colon, sigmoid colon, rectum and unspecified location.

### Characteristics associated with the longest DI and the start of investigation through the emergency department

When examining the likelihood of having the longest DI for each cohort (Table 3), in 2012 none of the variables were associated with the longest DI. While in the 2018 cohort, being diagnosed with CRC in the right-side colon and living with lower economic standard were associated with the longest DI. However, only CRC in the right-side colon was associated with the longest DI in the adjusted model in the 2018 cohort. We also performed a logistic regression to examine the likelihood of an initial CRC investigation at the emergency department. In 2018, CRC in the right-side colon was the only characteristic associated with acute initiated investigations and this association remained after the adjustment for age and sex (OR 1.76, CI 1.17–2.65, *p* 0.007) (further data not presented).

**Table 3.** The association between characteristics of patients diagnosed with colorectal cancer (CRC) and the longest diagnostic interval (DI).

Cohorts	2012 Unadjusted OR (CI)	*p*-value	Adjusted OR (CI)	*p*-value	2018 Unadjusted OR (CI)	*p*-value	Adjusted OR (CI)	*p*-value
Age								
≤64 years	Ref		Ref		Ref		Ref	
65-72 years	1.01 (0.48–2.12)	0.981	1.06 (0.50–2.28)	0.878	0.56 (0.26–1.20)	0.134	0.51 (0.23–1.12)	0.095
73-79 years	1.10 (0.55–2.21)	0.786	1.12 (0.54–2.32)	0.761	0.99 (0.49–2.02)	0.976	0.84 (0.40–1.79)	0.655
≥80 years	1.63 (0.84–3.18)	0.149	1.58 (0.77–3.26)	0.213	1.75 (0.89–3.44)	0.105	1.50 (0.74–3.03)	0.259
Female	1.47 (0.91–2.38)	0.120	1.30 (0.78–2.15)	0.311	1.15 (0.72–1.84)	0.557	1.00 (0.60–1.68)	0.989
Shorter education	1.03 (0.55–1.92)	0.931	0.85 (0.44–1.65)	0.630	0.87 (0.51–1.51)	0.628	0.83 (0.47–1.50)	0.543
Lower economic standard	1.38 (0.80–2.39)	0.247	1.22 (0.67–2.20)	0.518	**1.77 (1.02–3.09)**	**0.044**	1.52 (0.82–2.84)	0.184
Longer distance to the hospital	1.06 (0.58–1.93)	0.852	0.99 (0.54–1.86)	0.996	0.98 (0.55–1.75)	0.941	0.83 (0.44–1.58)	0.577
CRC right side	1.30 (0.79–2.12)	0.302	1.21 (0.72–2.02)	0.475	**1.72 (1.06–2.78)**	**0.027**	**1.66 (1.00–2.76)**	**0.050**

Values are presented with an odds ratio (OR) and confidence interval (CI). Patients with the longest DI -The 20% with the longest DI was defined as a cut-off, DI ≥ 125 days in 2012 and DI ≥91 days in 2018. DI <125 days in 2012 and DI <91 days in 2018 were used as references. In 2012, n = 83 patients were included into the longest DI of N = 410 patients. In 2018, n = 89 patients were included into the longest DI of N = 434 patients.

**Age** – Divided into quartiles and presented in age groups, ≤64 years used as reference.

**Shorter education** - Completed elementary school or high school education. **Longer education** - Completed college or university education (reference).

**Lower economic standard** - Available family income in Swedish krona (SEK) / year (below 60% of median nationally each year), specifically <137300 SEK (<12 187 €) in 2012 and <153200 SEK (≈ <14 714 €) in 2018. **Higher economic standard** - Above lower economic standard in each cohort (reference).

**Longer distance** - The 20% with the longest distance to the hospital (57–264km). **Shorter distance** - <57 km to the nearest hospital (reference).

**CRC right side** - Ascending colon. **CRC all others** - Transverse colon, descending colon, sigmoid colon, rectum and unspecified location (reference).

**Adjusted** - for age, female, shorter education, lower economic standard, longer distance to hospital, and CRC right side.

## Discussion

The main finding was that the DI was reduced by approximately two weeks after the introduction of CPP. The reduction of DI was observed for most sociodemographic groups regarding age, sex, economic standard, level of education, distance to the hospital, and presented symptoms indicating an improvement in time to diagnosis following the introduction of CPP for CRC. However, in 2018, for the cases of CRC in the right-side colon, the reduction of DI was not observed and was associated with the longest DI and acute-initiated investigations. Finally, the DI was reduced for patients with CRC having the first contact with primary healthcare. We also observed a shorter patient interval after the introduction of CPP, though this has to be interpreted with caution due to missing information for many patients.

### Strengths and weaknesses

The major strength of this study is that the Swedish cancer register has a high coverage rate of reported cases (>98% in Sweden) diagnosed with CRC [25], which indicates high validity of data. Additionally, we compared DI in two relatively big cohorts and reviewed EHRs that include data on the process from primary to secondary healthcare in each county. Moreover, the data collection from EHR was provided by three physicians and two of them were involved in data collection in both cohorts, something that strengthens the validity and reliability of data. Furthermore, two of the authors were involved in managing data collection and one author confirmed all collected data a second time after initial collection and validation. We retrospectively collected data of patients diagnosed with CRC before and after CPPs and complemented with a review of the EHR. One limitation is that we lacked data concerning the number of examinations which limited the ability to assess how this could influence the DI. Furthermore, it is unlikely that the 16,000 inhabitants who were not possible to assess due to inaccessible EHR influenced the results considering that they only made up 2.5% of the population in the counties studied. Additional limitations of the study are that the results regarding patient interval should be interpreted with caution since there was a high proportion of missing cases (∼ 40%), potentially due to either a high recall bias or lack of documented reported patients’ first symptoms.

### Findings in relation to other studies

In line with our results, studies from other countries indicate an improvement of time to diagnosis after CPPs [15–17]. Our results showed a shortened DI after the introduction of CPPs mainly for patients with first contact in primary healthcare and in counties with longer DI before the introduction of CPPs. The CPPs aimed to reduce regional differences in time to diagnosis and our results indicate an improvement in the three included counties towards a DI closer to each other. The introduction of CPPs may have led to shortened DI and faster diagnosis of CRC in 2018 for patients starting their care trajectory in primary healthcare, while the DI and the number of cases starting in secondary healthcare remained similar as before the introduction of CPP.

Regardless of age, sex, education, economic standard, symptoms of cancer, and distance to the hospital, we found a trend of shorter DI in 2018 than in 2012. This reduction was observed for both the median of DI as well as the 75 percentile. However, our results revealed the same pattern concerning time to diagnosis for CRC in both 2018 as well as in 2012 where patients who were older, female, had shorter education, lower economic standard, longer distances to hospital, and without CPP-qualifying symptoms had longer DI. Reasons behind that are unknown but it indicates that we need other tools to improve DI for these groups. It is especially important since patients with CRC in the right-side colon are more often older and female with more advanced tumour stages when diagnosed [5]. Other studies have also shown that socioeconomic factors and distance to healthcare are associated with worse survival and more advanced CRC at diagnosis, which could indicate longer time to diagnosis [8–11]. Yet, our results show a reduction in DI within most sociodemographic groups. Furthermore, in the unadjusted model in 2018, long DI (≥91 days) was associated with both low economic standard and CRC in the right-side colon, while only the latter remained in the adjusted model. While we did not find an association between income, age and DI, some other studies have found association with income [12] and age [12,28].

When exploring the association between tumour localisation and DI, after the introduction of CPP, the DI was not shortened for patients with cancer in the right-side colon while the DI was reduced for cases with cancer localised in the rest of the colon and rectum. However, as already known, CRC in the right-side colon is more challenging to identify as it often presents with diffuse non-specific symptoms why often diagnosed in advanced stages leading to a worse prognosis [5,6]. Our results also indicate that CPP for CRC seems to reduce DI for patients with CRC in the left part of the colon and rectum since these patients more often seek care presenting alarm symptoms (which CPPs focus on) such as rectal bleeding and blood in stool [29]. However, our results showed that patients with cancer in the right-side colon were associated with longer DI, acute admissions, and the start of investigation at the hospital. This indicates that CPPs generally do not improve the time to diagnosis for all patients and specifically not for those presenting non-alarming, diffuse non-specific symptoms and this challenge remain. Similarly, others have also found that CPPs seem to promote specific alarm symptoms and even prolong DI for patients with more difficult non-specific symptoms [21,29] and might contribute to a higher risk of crowding out effects for other serious diseases. Additionally, some patients in this study had a very long DI, e.g. twelve patients in 2012 and three in 2018 had a DI over 500 days, and two of these patients refused further investigation. Others have found that increased time to diagnosis is related to the patient’s perceptions of their symptoms as benign and not serious as well as the healthcare systems’ use of non-urgent referrals [12]. Another reason is a lower likelihood for patients to be referred to a CPP and thereby longer time to diagnosis when primary care physicians interpret symptoms as vague and not as alarm symptoms [21]. Our findings as well as those of others suggest that CPP does not contribute to earlier diagnosis for all patients.

## Conclusion and implications

The DI for CRC, both the median and the 75 percentile, was reduced overall and within almost all studied sociodemographic groups after the introduction of CPPs in northern Sweden. This association indicates that CPPs may have contributed to the improvement of DI for patients with CRC and particularly for those initially seeking primary healthcare. The evidence for the clinical importance of reduced DI found in the present study is uncertain, however, a reduced DI might improve the patient’s experience. Meanwhile, DI remained unchanged for patients with CRC in the right-side colon who still more often started their diagnostic process through acute referrals and admissions to the emergency room. This indicates that CPP does not facilitate improvement in DI and detection of tumours for patients presenting with non-specific symptoms often associated with CRC in the right-side colon. Moreover, some differences remained within the sociodemographic groups and need to be further researched. Even though the DI was reduced in general as well as for most groups after the introduction of the CPPs, this was not the case for all patients. Our results indicate that standardised care pathways are not an optimal solution to identify CRC among patients who are initially presenting with diffuse and non-specific symptoms. Thus, other solutions are needed to optimally manage patients having symptoms that do not fit into CPPs package for CRC. Therefore, a higher awareness of the fact that right-sided CRC is slowly growing and often presents with diffuse symptoms is important, especially in primary healthcare since most patients start their care pathway there.

## Data Availability

Due to the nature of this research, participants of this study did not agree for their data to be shared publicly, so supporting data is not available.
